# Sedentary behavior and neck pain in children and adolescents; a systematic review and meta-analysis

**DOI:** 10.34172/hpp.2022.31

**Published:** 2022-12-10

**Authors:** Sadegh Baradaran Mahdavi, Sadegh Mazaheri-Tehrani, Roya Riahi, Babak Vahdatpour, Roya Kelishadi

**Affiliations:** ^1^Department of Physical Medicine and Rehabilitation, School of Medicine, Isfahan University of Medical Sciences, Isfahan, Iran; ^2^Child Growth and Development Research Center, Research Institute for Primordial Prevention of Non-communicable Disease, Isfahan University of Medical Sciences, Isfahan, Iran; ^3^Student Research Committee, Isfahan University of Medical Sciences, Isfahan, Iran; ^4^Department of Epidemiology and Biostatistics, School of Public Health, Isfahan University of Medical Sciences, Isfahan, Iran

**Keywords:** Neck pain, Sedentary behavior, Screen time, Cell phone use, Meta-analysis

## Abstract

**Background:** Sedentary behavior (SB) is considered a risk factor for musculoskeletal pain. We aimed to explore the association of sedentary behavior indicators with neck pain among children and adolescents.

**Methods:** A comprehensive review was performed in different databases until the end of January 2022. Odds ratios (ORs) with 95% confidence intervals were used as desired effect sizes to evaluate the association between prolonged screen time or mobile phone (MP) usage and neck pain risk.

**Results:** Among 1651 records, 15 cross-sectional studies were included in the systematic review, and 7 reports were included in the meta-analysis. Our results suggested a significant relationship between prolonged MP use and neck pain (OR=1.36, 95% CI=1.001–1.85, I^2^=40.8%, *P* value for heterogeneity test=0.119). Furthermore, a marginally insignificant association was found between prolonged screen time and neck pain (OR=1.13, 95% CI=0.98–1.30, I^2^=60.3%, *P* value=0.01); however, after sensitivity analysis and removing one study, this association became significant (OR=1.30, 95% CI=1.03–1.64). Moreover, a significant association between prolonged sitting time and neck pain was reported in two studies.

**Conclusion:** Available good-quality evidence reveals a significant mild association between sedentary behavior and the risk of neck pain among children and adolescents. However, longitudinal studies with objective measurement tools are warranted. In particular, potential preventive educational programs are suggested for pediatrics to reduce sedentary behavior and neck pain.

## Introduction

 Musculoskeletal pain (MSP) is a worldwide problem affecting people of all ages in both sexes. It is one of the major reasons for disability-adjusted life years.^1,2^ Neck pain (NP) is one of the most common MSPs, with a 27 per 1000 people prevalence rate. The United States spent approximately $134.5 billion on low back and NP in 2016.^3^ NP could be due to various reasons, including the involvement of nerve roots, muscles, joints, ligaments, or intervertebral discs. Most of the time, there is no underlying systemic disorder; thus, it is included in MSPs.^4^ Based on the World Health Organization (WHO) reports, NP is the 8^th^-ranked reason for years lived with disability in adolescents.^5^ Moreover, evidence suggests that persistent MSPs during childhood increase the risk of developing chronic MSPs in adulthood.^6-8^ Nevertheless, MSPs in adolescents have not been as extensively focused on as in adults.

 Sedentary behaviors (SBs) involve activities with very low energy expenditure that are mostly performed in sitting or lying down positions, such as mobile phone (MP) use, computer use, TV watching, video gaming, and prolonged sitting.^9,10^ There is confirmed data about the association between SB and non-communicable diseases like cardiovascular disease and cancer.^11,12^ Besides, SBs are one of the items previously correlated to NP in adults and adolescents and are suggested to be an essential factor for differences in NP prevalence in populations.^13-15^ However, some studies did not suggest any significant association between SB and NP. For instance, Dianat et al^16^ reported no remarkable association between computer use, playing video games, and watching TV with NP history in the past month. Similarly, Silva et al^17^ did not report a significant relationship between MP and computer use with NP history in the last six months.

 With more innovations in technology, an increase in SBs is unavoidable. Thus, to summarize the available evidence and address the controversies, we aimed to assess the relationship between different types of sedentary behaviors and NP, among children and adolescents, through a systematic literature review and meta-analysis.

## Methods

###  Search strategy

 We carried out the current study based on PRISMA 2020 statement.^18^ The PROSPERO code for the protocol of this study is CRD42022313079. The systematic search was conducted in biomedical databases, including Web of Science, Scopus, PubMed, Embase, and Google Scholar, for available records until January 2022. Based on our research question, the search strings were made by combining equivalents of sedentary behavior (including screen time and sitting time) on the one hand and neck pain on the other in the biomedical databases. The search line for each mentioned database is shown in Supplementary file 1.

###  Eligibility criteria and study selection

**Figure 1:**
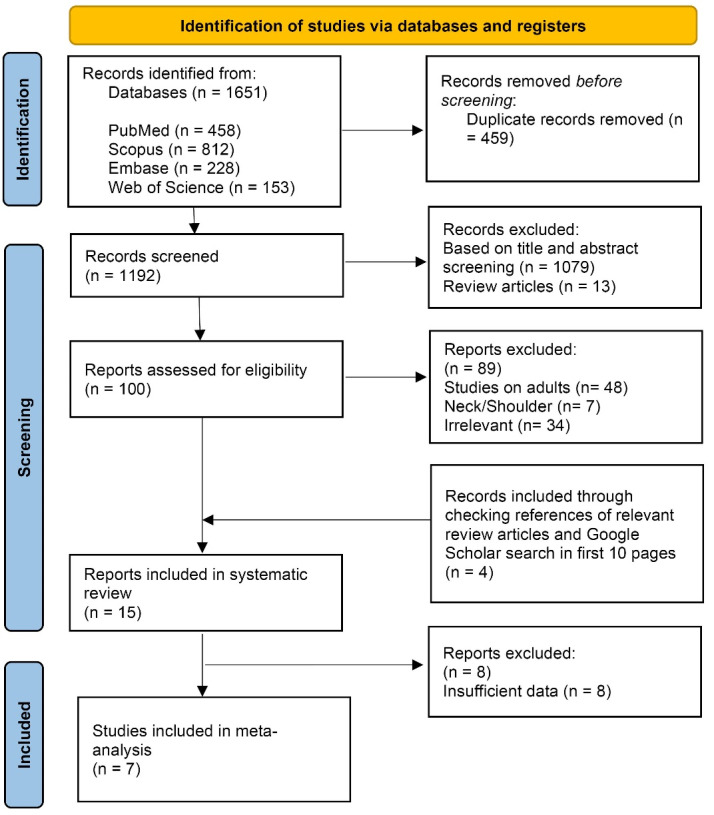
PRISMA flow diagram

 After removing the duplicates, two independent reviewers (SMB and SM-T) reviewed and screened the records based on the title, abstract, and full text. Meanwhile, conference proceedings, books, letters, and reviews were deleted. Additionally, the references of related review articles were checked to find undetected appropriate studies. The third reviewer (RK) resolved any disagreement related to eligible records. Only English-language and full-text articles (on human subjects) were included. Figure 1 shows the process of eligibility assessment.

###  Inclusion criteria

 All observational research (cohort, case-control, and cross-sectional studies) on children or adolescents that assessed the relationship between different forms of SBs (screen time and sitting time) and NP were eligible to be included without the restriction of race, gender, and publication date.

###  Exclusion criteria

 Studies with the following criteria were excluded: experimental research, studies on neck/shoulder pain, studies on cervical radicular pain, studies that assessed neck disability index, and studies with insufficient data.

###  Data extraction 

 Two independent reviewers (SBM, SM-T) performed data extraction from included articles. First author’s name, study design, publication date, study participants, sample size, gender, age range, type of SB, NP assessment tool, and a summary of main findings were extracted.

###  Quality assessment

 The National Institute of Health (NIH) quality assessment tool was utilized to evaluate the quality of included records.^19^ The scale consists of 14 items for cross-sectional and cohort studies. Based on the mentioned tool, the quality of articles classifies as good, fair, and poor.

###  Data synthesis

 For the meta-analysis, studies that investigated computer, tablet, and TV watching, as well as video gaming, were categorized as screen time. According to available data, the odds ratios (ORs) with 95% confidence intervals (CIs) were used as the effect sizes to evaluate the relationship between prolonged screen time or MP use and the risk of NP among children and adolescents. The random-effects model using DerSimonian and Laird was used for data analysis. Corresponding forest plots were made for both exposure factors. The heterogeneity of records was evaluated by Cochrane’s Q statistic and the inconsistency index (I^2^-statistic). The leave-one-out method was utilized for sensitivity analysis.

 Moreover, the possible publication bias was assessed with funnel plots and was quantitatively examined by both Egger’s and Begg’s tests. We would use the trim-and-fill method to adjust the effect if there was publication bias. A two-sided *P* < 0.05 was considered significant in all cases. All analyses were conducted by STATA v12 (Stata Corporation, College Station, Texas, USA).

## Results

###  Study selection

 According to the initial search in the mentioned databases, 1651 records were recognized. After excluding duplications, 1192 papers were screened through the title and abstract. Then, 100 articles remained for further investigations via full text. Eventually, 15 articles were eligible for inclusion in the qualitative analysis, and 7 reports were included in the quantitative analysis. Figure 1shows the PRISMA flow diagram.

###  Study characteristics 

 All 15 included articles were in the cross-sectional design. The participants of all studies were school students (age range of 8- to 19-year-old), with nearly equal participants of both genders. These 15 studies explored 15512 participants from different years (from 2008 to 2022). The smallest sample size was 91 school students in Indonesia,^20^ and the largest one was 2750 from a study in Thailand.^21^ Included research was from different countries, including three in Brazil,^14,15,22^ two in Portugal,^17,23^ two in South Africa,^24,25^ and one study from Pakistan,^13^ Tunisia,^26^ Saudi Arabia,^27^ Thailand,^21^ Iran,^16^ Indonesia,^20^ Taiwan,^28^ and Bosnia and Herzegovina.^29^

 MP usage, computer usage, watching TV, and playing video games are the most frequent SBs that have been assessed in six,^13-15,17,20,23^ seven,^14,16,21,22,24-26^ five,^14,16,21,23,26^ and four^16,22,23,26^ articles respectively. Other SBs like tablet usage, e-devices usage, sitting and doing homework, phone talking and texting, and sitting duration in school have also been assessed.

 According to our search results, we divided the measured outcomes into screen time (computer usage, watching TV, and playing video games), MP usage, and sitting time (sitting duration in school and sitting time while doing homework). Based on the available data, we perform meta-analyses on studies evaluating the association between screen time or MP usage and NP.

 Studies used different methods, including Questionnaires, interviews, and one-item questions for NP assessment. Each study investigated the history of NP in various timeframes, from the last week to the last 12 months. Table 1 presents the complete information of included studies.

**Table 1:** Included studies in the systematic review

**Author, year**	**Country**	**Study design**	**Participants**	**Sample size**	**Gender (male%)**	**Age (year)**	**Sedentary behavior**	**Assessment of NP**	**Outcome **	**Main finding**	**QA**
Mandhwani et al 2022 ^13^	Pakistan	Cross-sectional	School-going children	385	50	11-15	MP usage	One-item question	NP in the past 12 months	MP usage associated with NP	Fair
De Vitta et al 2021^* 14^	Brazil	Cross-sectional	High school students	1628	49	14-18	Watching TV, computer use, MP usage, tablet usage	Interview	NP in the past 12 months	NP was associated with computer use ( > 3 h/day), MP usage in standing position, MP usage ( > 3 h/day), tablet usage in both standing and sitting positions, and tablet usage ( > 3 h/day)	Good
de Brito Nunes et al. 2021 ^15^	Brazil	Cross-sectional	High school students	286	53.1	15-19	MP addiction	One-item question	Cervical pain in the last 7 days	MP addiction was associated with cervical pain	Good
Minghelli 2019^* 23^	Portugal	Cross-sectional	Elementary school students and high school students	304	47.4	10-17	MP usage, watching TV, console games	Interview	NP in the past 12 months	NP was associated with just MP usage	Good
Ben Ayed et al. 2019^* 26^	Tunisia	Cross-sectional	Secondary school grade students	1221	40.3	12-18	Computer use, watching TV, playing videogames	Questionnaire	NP in the past 3 months	NP was associated with just computer use ( > 4 h/week)	Good
Alzaid et al. 2018 ^27^	Saudi Arabia	Cross-sectional	Children	2435	58	< 18	e-devices usage	Questionnaire	NP	NP was associated with time spent on e-devices	Fair
Keeratisiroj and Siritaratiwat 2018 ^21^	Thailand	Cross-sectional	Primary and secondary school children	2750	50	10-19	Computer use, sitting and doing homework, watching TV	Questionnaire	NP in the past 12 months	NP was associated with computer use and sitting and doing homework	Good
Dianat et al 2017^* 16^	Iran	Cross-sectional	School children	1611	46.6	11-14	Computer use, playing video games, watching TV	Questionnaire	NP in the past month	There is no association	Good
Silva et al. 2017^* 17^	Portugal	Cross-sectional	High school students	969	48.2	13-19	MP usage, computer usage	Questionnaire	NP in the last 7 days	There is no association	Good
Widhiyanto et al 2017 ^20^	Indonesia	Cross-sectional	School students	91	41.8	NR	Duration of MP usage	Questionnaire	NP	Duration of MP usage was associated with NP	Fair
Yang et al 2016^* 28^	Taiwan	Cross-sectional	Junior college students	302	60.3	NR	Phone talking, texting,	Questionnaire	NP in the past 6 months	There is no association	Good
Silva et al 2015 ^22^	Brazil	Cross-sectional	High school students	961	38	14-19	Computer use, playing video games, total time spent on e-devices	Questionnaire	Cervical pain in the past 6 months	Cervical pain was associated with computer usage ( > 3 h/day), video gaming ( > 1 h/day), and the total time of use ( > 4 h/day)	Good
Azabagic et al 2016 ^29^	Bosnia and Herzegovina	Cross-sectional	Primary school children	1315	49.5	8-12	Sitting duration in the school and during homework	Questionnaire	Acute NP	NP was associated with sitting duration in the school and during homework	Good
Mafanya and Rhoda 2011 ^25^	South Africa	Cross-sectional	High school students	181	53.6	14-18	Computer usage at school and elsewhere	Questionnaire	NP in the past month	NP was associated with computer usage elsewhere	Fair
Smith et al 2008^* 24^	South Africa	Cross-sectional	High school students	1073	35.1	14-18	Computer usage ( > 8.5 h/week)	Questionnaire	NP in the past month	NP was associated with high hours of computer usage	Good

Abbreviations; MP: mobile phone, NP: neck pain, QA: Quality assessment, e-devices: electronic devices, NR: not reported. ^*^ Included articles in the meta-analysis.

 According to the NIH quality assessment tool, among 15 included articles in the systematic review, 11 studies were qualified as good quality, and 4 studies as fair quality. All included papers in the meta-analysis had good quality. Table 2reveals the quality assessment of included studies in detail.

**Table 2:** Quality assessment of included studies in the systematic review

**Study**	**Questions of NIH quality assessment tool for cohort and cross-sectional studies** ^a^														**Summary quality**
	**1**	**2**	**3**	**4**	**5**	**6**	**7**	**8**	**9**	**10**	**11**	**12**	**13**	**14**	
Mandhwani et al 2022 ^13^	Yes	Yes	Yes	Yes	No	No	No	Yes	No	No	Yes	Yes	NA	No	Fair
de Vitta et al 2021 ^14^	Yes	Yes	Yes	Yes	No	No	No	Yes	Yes	No	Yes	Yes	NA	Yes	Good
de Brito Nunes et al 2021 ^15^	Yes	Yes	Yes	Yes	No	No	No	Na	Yes	No	Yes	Yes	NA	Yes	Good
Minghelli 2019 ^23^	Yes	Yes	Yes	Yes	No	No	No	Yes	No	No	Yes	Yes	NA	Yes	Good
Ben Ayed et al 2019 ^26^	Yes	Yes	Yes	Yes	Yes	No	No	Yes	Yes	No	Yes	Yes	NA	Yes	Good
Alzaid et al 2018 ^27^	Yes	Yes	Yes	Yes	No	No	No	Yes	No	No	No	Yes	NA	Yes	Fair
Keeratisiroj and Siritaratiwat 2018 ^21^	Yes	Yes	Yes	Yes	Yes	No	No	Yes	No	No	Yes	Yes	NA	Yes	Good
Dianat et al 2017 ^16^	Yes	Yes	Yes	Yes	No	No	No	Yes	Yes	No	Yes	Yes	NA	Yes	Good
Silva et al 2017 ^17^	Yes	Yes	Yes	Yes	No	No	No	Yes	Yes	No	Yes	Yes	NA	Yes	Good
Widhiyanto et al 2017 ^20^	Yes	No	No	Yes	Yes	No	No	Yes	No	No	No	Yes	NA	No	Fair
Yang et al 2016 ^28^	Yes	No	Yes	Yes	No	No	No	Yes	Yes	No	Yes	Yes	NA	No	Good
Azabagic et al 2016 ^29^	Yes	Yes	Yes	Yes	No	No	No	Yes	Yes	No	Yes	Yes	NA	No	Good
Silva et al 2015 ^22^	Yes	Yes	Yes	Yes	Yes	No	No	Yes	Yes	No	Yes	Yes	NA	Yes	Good
Mafanya and Rhoda 2011^25^	Yes	No	Yes	Yes	No	No	No	Yes	No	No	Yes	Yes	NA	Yes	Fair
Smith et al 2008 ^24^	Yes	Yes	Yes	Yes	Yes	No	No	Yes	Yes	No	Yes	Yes	NA	Yes	Good

^a^Available online at: https://www.nhlbi.nih.gov/health-topics/study-quality-assessment-tools. NA, Not applicable.

###  Main findings of meta-analysis for prolonged screen time

**Figure 2:**
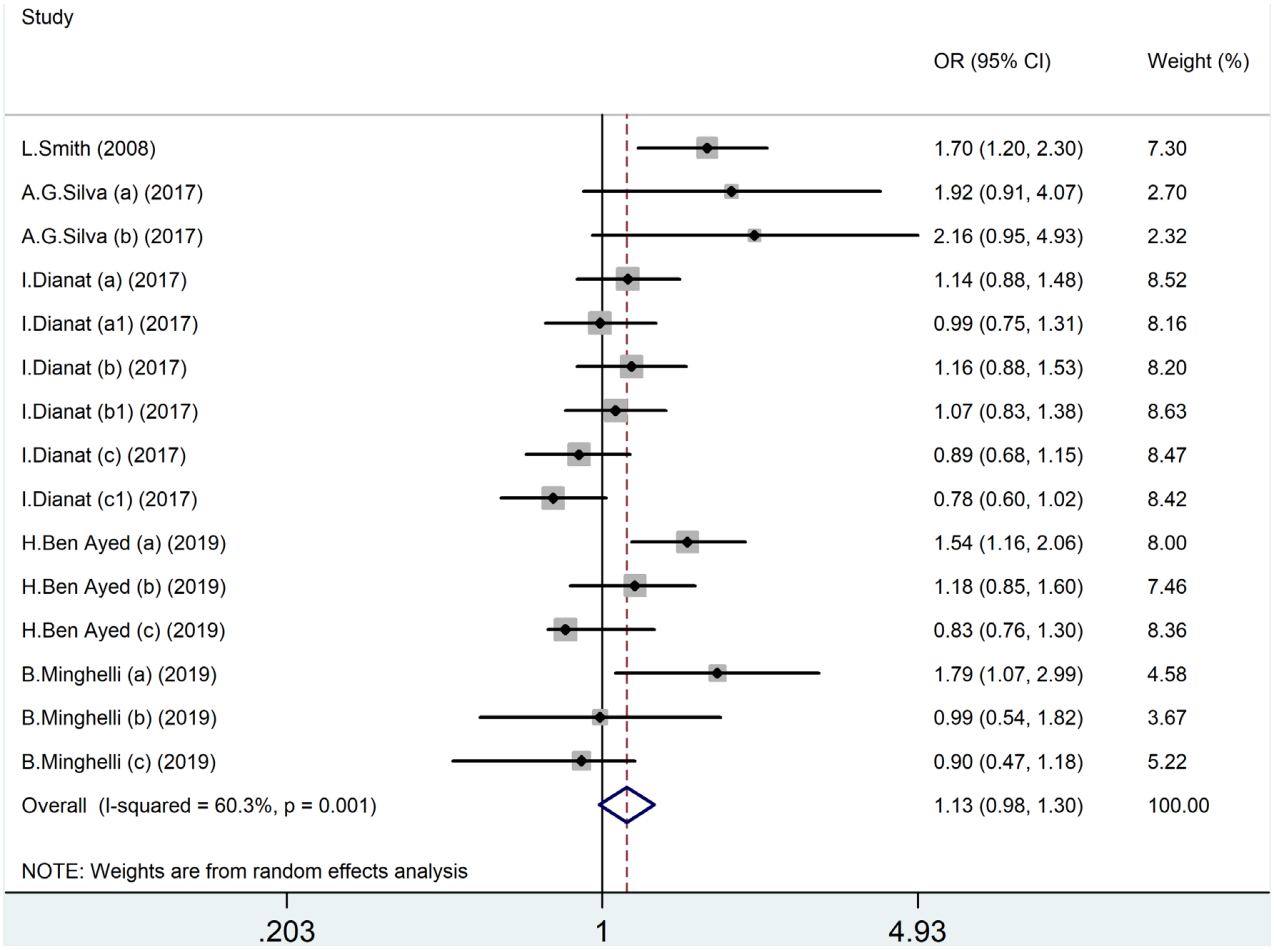
The pooled odds ratio for the association between prolonged screen time and neck pain among children and adolescents

 Results of the meta-analysis based on the crude odds ratio as desired effect size for the relationship between prolonged screen time and NP are shown in Figure 2. The pooled ORs by random effect model indicated no significant relationship between prolonged screen time and NP (OR = 1.13, 95% CI = 0.98–1.30), with significant heterogeneity (I^2^ = 60.3%, *P* value = 0.01).

**Figure 3:**
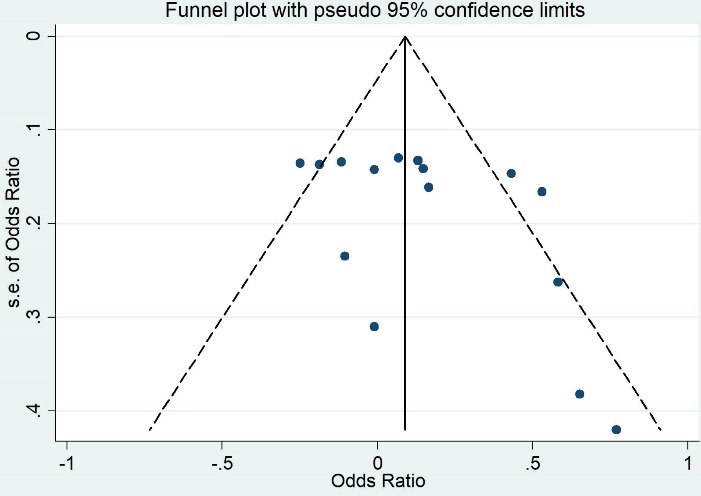
Funnel plot for publication bias of prolonged screen time and risk of neck pain

 Both Egger and Begg’s tests showed no evidence of publication bias (the *P* value of Egger’s test = 0.66, and the *P* value of Begg’s test = 0.60) (Figure 3).

###  Main findings of meta-analysis for prolonged MP use

**Figure 4:**
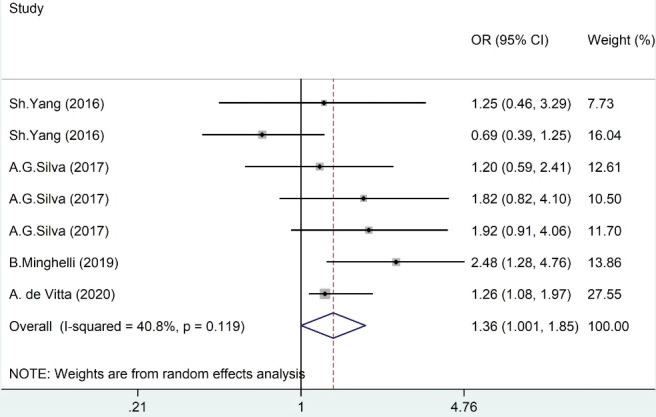
The pooled odds ratio for the association between prolonged MP use and neck pain among children and adolescents

 Results of the meta-analysis based on the adjusted odds ratio as desired effect size for the association between prolonged MP use and NP are summarized in Figure 4. The pooled ORs by random effect model showed that prolonged MP use was a significant risk factor for NP (OR = 1.36, 95% CI = 1.001–1.85), with non-significant heterogeneity (I^2^ = 40.8%, *P* value = 0.119).

**Figure 5:**
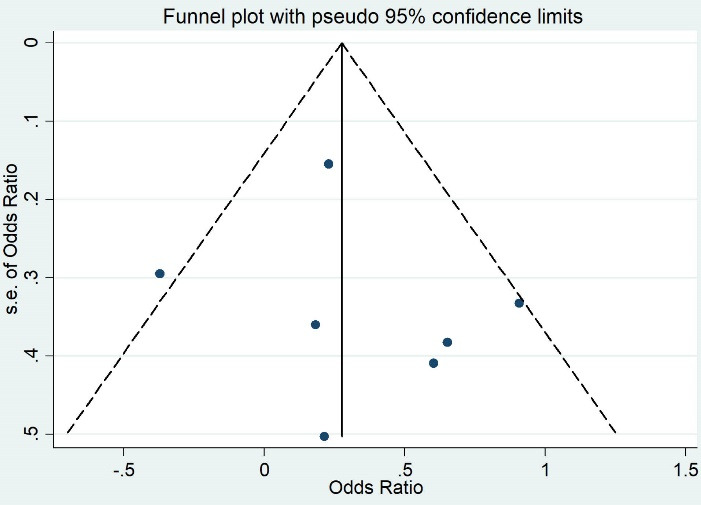
Funnel plot for publication bias of prolonged MP use and risk of neck pain

 Both Egger and Begg’s tests showed no publication bias (the *P* value of Egger’s test = 0.59, and the *P* value of Begg’s test = 0.45) (Figure 5).

###  Sensitivity analysis 

 Sensitivity analyses revealed no significant differences between the before-after sensitivity pooled OR for the association between prolonged screen time and NP after excluding Silva et al,^17^ Ben Ayed et al,^26^ Minghelli et al,^23^ and Smith et al^24^ studies. However, results indicated a significant change between before-after sensitivity pooled OR for the association between prolonged screen time and NP after excluding Dianat et al study^16^ (OR = 1.30; 95% CI, 1.03–1.64).

###  Prolonged sitting time and NP

 Two studies explored the association between prolonged sitting time and NP. Keeratisiroj and Siritaratiwat indicated that prolonged sitting and doing homework was associated with NP in the last 12 months among school children in Thailand.^21^ Another study on school children in Bosnia and Herzegovina demonstrated a significant relationship between sitting duration in school and during homework with acute NP.^29^ However, research on sitting time and neck pain in children is scarce and needs extra focus.

## Discussion

 This review investigated the relationship between different types of SBs and NP in adolescents and children. Results of the meta-analysis indicated a marginally insignificant association of screen time with NP. However, this association was significant after excluding one study in the sensitivity analysis (OR = 1.30). Moreover, we found a remarkable association between MP use and NP (OR = 1.36).

 Some controversies exist in reviewing the literature. Dianat et al^16^ demonstrated no significant relationship between computer use, watching TV, and playing video games with NP in the last month. Alternatively, Silva et al^17^ suggested no significant relationship between MP and computer use with NP in the last seven days. These findings are in contrast with other studies,^14,21-23,26^ and that might be, on the one hand, because of the unknown level of the certainty of the information given about MSPs and the amount of time devoted to SBs, and on the other hand, differences in the time of neck pain history.

 Nowadays, the time spent in SBs among adolescents and children has increased dramatically because of innovations in technology.^30^ Besides, during the current COVID-19 pandemic, SBs increased in all age groups, including adolescents and children.^31^ SBs in adolescents are demonstrated to have an association with an increased risk of a health crisis, including increased body mass index,^32^ higher blood pressure,^33^ and even psychosomatic health problems such as headaches, abdominal pains, irritability, difficulty falling asleep and feeling nervous.^34^

 NP is a widespread and multifactorial public health crisis. Several neck structures, including discs, ligaments, joints, muscles, and nerves, might be the source of pain.^35^ Different pathologies could represent NP, such as tumors, congenital disorders, infections, and inflammatory diseases. However, most conditions have no systemic complications and are named MSPs.^36^ Recently, there has been a rise in adolescents complaining of NP.^37^ “Text Neck” syndrome is a newly emerged term used to describe the NP because leaning forward for prolonged periods, looking at the MP, tablet, and other e-devices while texting. Finally, prolonged neck flexion results in the “text neck”.^38^ Based on the text neck concept, higher weight is put on the spine with more flexion in the neck.^39^ The relation between text neck and NP is questionable. Some studies support the idea of an association of text neck with NP among adolescents^5,40,41^; however, some others did not find any relationship.^42,43^

 Many researches have suggested obesity as a risk factor for NP in adolescents.^44,45^ Obesity is an important health problem with increasing rates among children and adolescents with a sedentary lifestyle.^46^ However, there is conflicting evidence on the association of obesity with NP. A retrospective cohort study on primary school children reported a significant association between obesity and NP,^47^ in line with a survey conducted by Martínez-Romero et al^48^ and Dianat et al^16^ However, some other studies did not report any association.^49,50^ On the other hand, a cohort research with two years of follow-up on 848 school children in Denmark demonstrated a higher incidence of overweight among children with pain in the spinal column. The relative risk for becoming overweight was reported (RR = 5.3, 95% CI = 3.98–7.58) for children with spinal pain and (RR = 1.6, 95% CI = 0.19–5.45) for children without.^51^

 Physical activity is a possible protective factor against developing NP in adolescents.^52,53^ Lower physical activities are associated with higher SBs^54^; thus, they could be associated with NP. Enough physical activity diminishes spinal pain and NP by reducing muscle tension, decreasing pressure on vertebral discs, and increasing blood circulation.^55^ However, a systematic review demonstrated no significant relationship between physical activity and NP in children.^56^

 One crucial factor that is associated with higher rates of MSPs in school-aged children is heavy-weight school bags. The average weight of a school bag should not exceed 10% of the body weight; otherwise, irreparable consequences on the spinal cord are unavoidable.^57^ Thus, according to their child, parents’ knowledge about a standard-weight backpack should increase.^58^

 All SBs are approximately indoor activities, which reduce outdoor physical activity and, as a result, sun exposure. Thus, limited sunlight exposure causes lower serum vitamin D levels in a sedentary lifestyle.^59,60^ On the other hand, investigations demonstrated that vitamin D deficiency increases MSPs rates among children^61^ and also showed a significant decrease in MSPs of vitamin D deficient children prescribed vitamin D for six months.^62^ Given the information, vitamin D deficiency might be an underlying mechanism for NP in groups with higher SBs.^63^

 Most children and adolescents are unaware of the long-term effect of MSPs because there are no severe short-term effects. On the other hand, adolescents are the greatest users of e-devices. Thus, paying attention to this group is essential to reduce their future disabilities and increasing life expectancy.^5^ Based on the latest WHO guideline on physical activity and sedentary behavior, children and adolescents is recommended for at least one hour of moderate-to-vigorous intensity physical activity per day. Moreover, a strong association was observed between lower SBs and a lower risk of future non-communicable diseases.^64,65^

###  Limitations of data synthesis 

 First, all the included studies used self-reported data; thus, various factors could affect the certainty level of collected data. Second, included studies assessed the associations among school children, and it might be questionable whether the pooled results could be generalized to the other groups of adolescents. Third, our results indicated a mild relationship between SBs and NP, possibly because of the limited number of included studies or the moderating effects of other factors related to children’s neck pain. Moreover, all included articles in this study were in a cross-sectional design. Further longitudinal studies will demonstrate the relationship and causality effect more accurately.

###  Strengths of the study

 First, there is limited data on neck pain and sedentary behavior among children and adolescents, and the current work is the first review and meta-analysis in this field. Second, all included articles had acceptable quality and no publication bias.

## Conclusion

 Based on available good-quality evidence, MP use is associated with an increased risk of NP. Moreover, a marginally insignificant association between screen time with NP is observed. However, research on sitting time and neck pain in children is scarce and needs extra focus. It seemed necessary that the effect of sedentary behaviors such as screen time be assessed in different populations, especially after the COVID-19 pandemic, which increased the sedentary behaviors among adolescents, to determine the effect more accurately. Moreover, it is recommended that further studies aim to assess NP using unified objective tools validated across different cultures.

## Author Contributions

**Conceptualization:** Roya Kelishadi, Sadegh Baradaran Mahdavi, Sadegh Mazaheri-Tehrani.

**Data curation:** Sadegh Mazaheri-Tehrani, Roya Riahi, Sadegh Baradaran Mahdavi.

**Formal Analysis: **Roya Riahi.

**Investigation:** Sadegh Baradaran Mahdavi, Sadegh Mazaheri-Tehrani.

**Methodology: **Roya Kelishadi, Sadegh Baradaran Mahdavi.

**Project administration: **Roya Kelishadi, Babak Vahdatpour.

**Software: **Roya Riahi, Sadegh Mazaheri-Tehrani.

**Supervision: **Roya Kelishadi, Babak Vahdatpour.

**Writing – original draft:** Sadegh Baradaran Mahdavi, Sadegh Mazaheri-Tehrani, Roya Riahi.

**Writing – review & editing: **Sadegh Baradaran Mahdavi, Sadegh Mazaheri-Tehrani, Roya Riahi, Roya Kelishadi, Babak Vahdatpour.

## Funding

 This research received no specific grant.

## Ethical Approval

 Not applicable.

## Competing Interests

 None to declare.

## Supplementary Files


Supplementary file 1 containsthe search line for each mentioned database.
