# Supplementary material for: Sedentary behavior and neck pain in children and adolescents; a systematic review and meta-analysis
Source: Health Promot Perspect. 2022 Dec 10;12(3):240–8. doi: 10.34172/hpp.2022.31 (PMC9808908; doi:10.34172/hpp.2022.31)
Supplement: Supplementary file 1 — containsthe search line for each mentioned database. [file hpp-12-240-s001.pdf]

Baradaran Mahdavi et al, **Health Promotion Perspectives,** 2022, 12(3), 240-248
doi: 10.34172/hpp.2022.31
https://hpp.tbzmed.ac.ir

Sedentary behavior and neck pain in children and adolescents; a systematic review and meta-analysis

Sadegh Baradaran Mahdavi[1,2,3], Sadegh Mazaheri-Tehrani[2,3*], Roya Riahi[2,4], Babak Vahdatpour[1], Roya Kelishadi[2*]

[1]Department of Physical Medicine and Rehabilitation, School of Medicine, Isfahan University of Medical Sciences, Isfahan, Iran
[2]Child Growth and Development Research Center, Research Institute for Primordial Prevention of Non-communicable Disease, Isfahan University of Medical Sciences, Isfahan, Iran
[3]Student Research Committee, Isfahan University of Medical Sciences, Isfahan, Iran
[4]Department of Epidemiology and Biostatistics, School of Public Health, Isfahan University of Medical Sciences, Isfahan, Iran

Supplementry file 1

Search string for PubMed

(("Neck Pain"[Mesh] OR "Neck Ache*" OR Neckache* OR Cervicalgia* OR Cervicodyni* OR "Cervical Pain*" OR "Posterior Cervical Pain*" OR "Posterior Neck Pain*" OR "neck pain*") AND ("Sedentary Behavior"[Mesh] OR "Screen Time"[Mesh] OR "Sedentary Lifestyle" OR "Physical Inactivity" OR "uncomfortable workstation" OR "bad work-habit*" OR "body posture" OR "ergonomic knowledge" OR "Sedentary Behavior*" OR "Screen Time" OR "daily computer usage" OR "working on a computer" OR "playing video game" OR "watching TV" OR sedentary OR "sitting time" OR "using mobile phone" OR "using smartphone"))

458 records (with no filter)

Search string for Web of Science

("Neck Ache*" OR Neckache* OR Cervicalgia* OR Cervicodyni* OR "Cervical Pain*" OR "Posterior Cervical Pain*" OR "Posterior Neck Pain*" OR "neck pain*") AND ALL FIELDS: ("Sedentary Lifestyle" OR "Physical Inactivity" OR "uncomfortable workstation" OR "bad work-habit*" OR "body posture" OR "ergonomic knowledge" OR "Sedentary Behavior*" OR "Screen Time" OR "daily computer usage" OR "working on a computer" OR "playing video game" OR "watching TV" OR sedentary OR "sitting time" OR "using mobile phone" OR "using smartphone")

153 records (with no filter)

Search string for Scopus

("Neck Ache*" OR neckache* OR cervicalgia* OR cervicodyni* OR "Cervical Pain*" OR "Posterior Cervical Pain*" OR "Posterior Neck Pain*" OR "neck pain*") AND ("Sedentary Lifestyle" OR "Physical Inactivity" OR "uncomfortable workstation" OR "bad work-habit*" OR "body posture" OR "ergonomic knowledge" OR "Sedentary Behavior*" OR "Screen Time" OR "daily computer usage" OR "working on a computer" OR "playing video game" OR "watching TV" OR sedentary OR "sitting time" OR "using mobile phone" OR "using smartphone")

812 records (Title-Abstract-Keywords)

Search string for Embase

('neck ache*' OR neckache* OR cervicalgia* OR cervicodyni* OR 'cervical pain*' OR 'posterior cervical pain*' OR 'posterior neck pain*' OR 'neck pain*') AND ('sedentary lifestyle' OR 'physical inactivity' OR 'uncomfortable workstation' OR 'bad work-habit*' OR 'body posture' OR 'ergonomic knowledge' OR 'sedentary behavior*' OR 'screen time' OR 'daily computer usage' OR 'working on a computer' OR 'playing video game' OR 'watching tv' OR sedentary OR 'sitting time' OR 'using mobile phone' OR 'using smartphone')

228 records (with no filter)
